# Development, Characterization, and Evaluation of Chi-Tn mAb-Functionalized DOTAP-PLGA Hybrid Nanoparticles Loaded with Docetaxel for Lung Cancer Therapy

**DOI:** 10.3390/pharmaceutics17020164

**Published:** 2025-01-25

**Authors:** Analía Castro, Álvaro Pittini, Nora Berois, Ricardo Faccio, Pablo Miranda, Álvaro W. Mombrú, Eduardo Osinaga, Helena Pardo

**Affiliations:** 1 Centro NanoMat, Instituto Polo Tecnológico de Pando, Facultad de Química, Universidad de la República, Canelones 91000, Uruguay; acastro@fq.edu.uy (A.C.); rfaccio@fq.edu.uy (R.F.); pmiranda@fq.edu.uy (P.M.); amombru@fq.edu.uy (Á.W.M.); 2 Laboratorio de Glicobiología e Inmunología Tumoral, Institut Pasteur de Montevideo, Montevideo 11400, Uruguay; apittini@pasteur.edu.uy (Á.P.); nberois@pasteur.edu.uy (N.B.); eosinaga@pasteur.edu.uy (E.O.); 3 Departamento de Inmunobiología, Facultad de Medicina, Universidad de la República, Montevideo 11800, Uruguay; 4 Cátedra de Física, Facultad de Química, DETEMA, Universidad de la República, Montevideo 11800, Uruguay

**Keywords:** lipid polymer hybrid nanoparticles, active targeting, cancer treatment, Tn antigen, PLGA, DOTAP

## Abstract

**Background/Objectives**: The focus of this study was to prepare and characterize docetaxel (DCX)-loaded lipid/polymer hybrid nanoparticles (LPHNps) functionalized with the monoclonal antibody (mAb) Chi-Tn for a potential active targeting approach in lung cancer treatment. **Methods**: We synthesized DOTAP-PLGA hybrid nanoparticles loaded with DCX and functionalized them with Chi-Tn mAb through a biotin–avidin approach. The physicochemical characterization involved dynamic light scattering, transmission electron microscopy, Raman spectroscopy, and atomic force microscopy. The in vitro and in vivo evaluations encompassed uptake studies, cell viability tests, and the assessment of tumor growth control in a lung cancer model. **Results**: The nanoparticles featured a hydrophobic PLGA core with 99.9% DCX encapsulation efficiency, surrounded by a DOTAP lipid shell ensuring colloidal stability with a high positive surface charge. The incorporation of PEGylated lipids on their surface helps evade the immune system and facilitate Chi-Tn mAb attachment. The resulting nanoparticles exhibit a spherical shape with monodisperse particle sizes averaging 250 nm, and demonstrate sustained drug release. In vitro uptake studies and viability assays conducted in A549 cancer cells show that the Chi-Tn mAb enhances nanoparticle internalization and significantly reduces cell viability. In vivo studies demonstrate a notable reduction in tumor volume and an increased survival rate in the A549 tumor xenograft mice model when DCX was encapsulated in nanoparticles and targeted with Chi-Tn mAb in comparison to the free drug. **Conclusions**: Therefore, Chi-Tn-functionalized LPHNps hold promise as carriers for actively targeting DCX to Tn-expressing carcinomas.

## 1. Introduction

Cancer is one of the most important public health problems of the 21st century. In particular, lung cancer was the leading cause of cancer-related morbidity and mortality worldwide in 2022 [1]. Despite the availability of various treatments, including surgery, radio-chemotherapy, molecular targeted therapy, and immunotherapy, the five-year survival rate for lung cancer remains below 20% in most countries [2]. Consequently, developing new therapies to improve survival rates is crucial.

Nanotechnology-based drug delivery systems have gained significant attention over the past decades due to their potential to reduce side effects, minimize toxicity, and enhance the efficacy of anticancer treatments [3]. The US Food and Drug Administration (FDA) has approved several nanocarriers for clinical use, with polymeric nanoparticles and liposomes being particularly promising because of their biocompatible and biodegradable properties [4]. Notable examples of commercially available liposomal antiproliferative drugs include Doxil and Daunoxome, which offer advantages such as increased solubility of the active compound, protection from adverse effects, high biocompatibility due to their similarity to biological membranes, prolonged drug release, and ease of surface modification. However, some drawbacks, such as poor storage stability, limit their broader application [5].

Polymeric nanoparticles are easier to prepare and exhibit high structural integrity, greater stability, and controlled release capability. Nevertheless, they have some disadvantages, including the need for organic solvents in their synthesis, low payload capacity, and moderate half-life in the bloodstream. One of the most widely used biopolymers, approved by the FDA and the European Medicines Agency (EMA), is poly(lactic-co-glycolic acid) (PLGA). PLGA is a versatile synthetic copolymer composed of glycolic acid and lactic acid units, the ratio of which determines some of its properties [6,7]. It is a biocompatible and biodegradable polymer, and it has been widely studied for the development of drug transport and release systems, such as small molecules, proteins, and other macromolecules, both for research and commercial developments.

To overcome the limitations of polymeric nanoparticles and liposomes while combining the favorable attributes of both nanosystems, a new generation of lipid/polymer hybrid nanoparticles (LPHNps) has been developed. LPHNps consist of a polymeric core surrounded by a lipid shell. The polymer constituent provides support to the hybrid structure while also being able to encapsulate both hydrophilic and hydrophobic drugs. The lipid portion helps avoid the rapid clearance of nanoparticles by the reticuloendothelial system (RES), can be chemically modified to enable targeted drug delivery, acts as a barrier to mitigate the leakage of entrapped drugs, and protects the core from degradation by preventing the diffusion of water into the inner core [8,9].

These nanoparticles, with lipid layers around a polymeric core, can be rapidly prepared using a single-step nanoprecipitation and self-assembly method to achieve high drug-encapsulation efficiency [10]. They are mainly composed of (1) PLGA as a hydrophobic core for encapsulating hydrophobic drugs, such as DCX, a chemotherapeutic agent with a wide spectrum of anti-tumor activity; (2) lipid 1,2-Dioleoyloxy-3-trimethylammonium-propane chloride (DOTAP-Cl) as a cationic lipid layer surrounding the PLGA core; and (3) 1,2-Distearoyl-sn-glycero-3-phosphoethanolamine-polyethylene glycol (DSPE-PEG) and DSPE-PEG-Biotin serve as polyethylene glycol (PEG) outer layer that incorporates into the lipid shell, extending the in vivo circulation half-life and offering functionalization groups for attaching targeting ligands.

Extensive research has revealed the critical role of glycan alteration in cancer therapy [11]. The identification of cancer-specific glycan biomarkers by monoclonal antibodies can be used to discriminate between tumor and normal cells, improving the specificity of tumor treatments [12]. The Tn antigen (GalNAc-O-Serine/Threonine) is considered a cryptic marker, typically not found in normal cells as it is masked by other sugar residues on mature glycan chains [13]. Tn is one of the most prevalent and specific tumor-associated antigens identified to date [14], making it a promising target for cancer diagnosis and immunotherapy. Based on the specificity of murine mAb 83D4 for the Tn antigen generated by our group [15], we produced a chimeric mouse/human antibody that retains the Tn binding activity [15]. This chimeric anti-Tn mAb (Chi-Tn) selectively binds to a diverse range of carcinomas without exhibiting any affinity for normal tissues [16]. It has also been shown to internalize cancer cells and deliver cytotoxic drugs actively [17]. To the best of our knowledge, there is currently no study that has yet developed cationic LPHNp functionalized with anti-Tn mAb.

In previous work, we developed chitosan nanocapsules loaded with DCX and functionalized with Chi-Tn antibodies [18]. However, these systems have certain limitations for broader use, particularly because chitosan is not a generally FDA-approved polymer for human applications. This distinction in composition highlights a significant innovation, as PLGA-based nanoparticles offer enhanced biocompatibility and greater potential for clinical translation compared to chitosan-based systems.

Considering the potential of LPHNp and the promising results previously obtained with DCX-loaded chitosan nanocapsules functionalized with Chi-Tn mAb [18], we propose that PLGA-DOTAP hybrid immunonanoparticles could serve as a novel and enhanced drug delivery platform for lung cancer treatment. In this study, we developed, characterized, and evaluated a novel DCX-loaded DOTAP-PLGA hybrid nanoparticle conjugated with Chi-Tn mAb as a potential cancer treatment. This formulation demonstrated high drug encapsulation efficiency and the active targeting of tumor cells, exhibiting enhanced anti-tumor activity in both in vitro and in vivo human lung cancer models.

## 2. Materials and Methods

### 2.1. Materials

Poly(d,l-lactic-*co*-glycolic acid), PLGA, 50:50 Resomer RG 502 H, molecular weight (Mw) 7000–17,000 g/mol, was purchased from Evonik (Buenos Aires, Argentina). The cationic lipid 1,2-Dioleoyl-3-trimethylammonium-chloride (DOTAP-Cl) was obtained from Lipoid (Steinhausen, Switzerland). Docetaxel, with a purity greater than 99%, was acquired from Alfa Aesar (Tewksbury, MA, USA). Crodamol GTCC, a blended ester made from caprylic and capric acids, was sourced from Croda (Buenos Aires, Argentina). 1,2-Distearoyl-sn-glycero-3-phosphoethanolamine-Poly-ethylene glycol (DSPE-PEG, Mw 2 KDa) and DSPE-PEG-Biotin (Mw 2 KDa) were purchased from Broadpharma (San Diego, CA, USA). Poloxamer 188 and Avidin from egg white (≥10 units/mg protein) were purchased from Sigma Aldrich (St. Louis, MO, USA). All the other reagents used were of analytical grade.

### 2.2. Cells Lines and Animals

A549 cells (purchased from American Type Culture Collection—ATCC, Manassas, VA, USA, catalog number CCL-185/RRID:CVCL_0023), derived from human lung carcinoma, were grown in Dulbecco’s modified Eagle’s Medium enriched with 10% fetal bovine serum and 1% glutamine. The cells were incubated at 37 °C in a humidified atmosphere containing 5% CO_2_.

Twenty-five female mice (*Mus musculus nude*) (26 ± 2 g, 6–8 weeks old) were bred by the Laboratory Animal Biotechnology Unit of the Institute Pasteur of Montevideo, Uruguay. All the animal experiments were conducted in accordance with the Ethics Committee on Animal Use at the Institut Pasteur of Montevideo (approval number 003-22), following the ARRIVE guidelines. The animals were housed in a controlled environment free of specific pathogens (SPF), with temperatures maintained between 19 and 21 °C, and a 14 h light/10 h dark cycle. The mice were provided with sterile food and water ad libitum. In this study, the only procedures performed on the mice were subcutaneous and intraperitoneal injections, which caused minimal discomfort, so anesthesia was not required. At the conclusion of the experiment, the mice were euthanized using 100% CO_2_.

### 2.3. Preparation of LPHNps

The preparation of nanoparticles was carried out through the self-assembly of lipid and polymeric components using the nanoprecipitation method resulting from the modification and combination of the protocols reported by Bose et al. [19], Dai et al. [20], and Klippstein et al. [21]. Specifically, 100 mg of PLGA was dissolved in 5.4 mL of acetone, which was then mixed with 0.5 mL of ethanol containing 7.5 mg of DOTAP, 5 mg of DSPE-PEG, 2.5 mg of DSPE-PEG-Biotin, and 125 µL of crodamol. This organic phase was divided into two equal portions. To one of the parts, 500 µL of a DCX stock solution (40 mg/mL in acetone) was added, resulting in drug-loaded nanoparticles (LPHNp-DCX), while the other half was added with 500 µL of acetone to subsequently obtain empty nanoparticles (LPHNps).

On the other hand, 50 mg of poloxamer 188 was dissolved in 10 mL of water; this aqueous phase was also divided into equal portions. Both were placed on heating plates until reaching a final temperature of 50 °C. Subsequently, the organic phase was slowly added dropwise to its respective aqueous phase under vigorous stirring. The resulting suspension was stirred for at least 2 h to allow self-assembly and ensure organic solvent removal. Finally, if necessary, the volume was adjusted to 5 mL. This way, nanoparticles with a final concentration of 4 mg of DCX/mL were obtained.

Fluorescent nanoparticles were prepared in the same manner as those loaded with DCX, replacing DCX with the fluorescent probe Coumarin-6 (adding 1 mL of an ethanolic solution of coumarin with a concentration of 0.2 mg/mL).

### 2.4. Functionalization of LPHNp with Chi-Tn mAb

LPHNp-Chi-Tn mAb was prepared by a two-step conjugation process involving pegylated nanoparticles and biotinylated Chi-Tn mAb utilizing the avidin–biotin interaction. In the first step, 10.8 µL of an avidin solution (2 mg/mL) was added to 2 mL of LPHNp. The mixture was then shaken in the dark for 1 h at room temperature. Following this, 48 µL of a biotinylated Chi-Tn mAb solution (1 mg/mL) was incorporated into the mixture, and shaking continued overnight. After these two incubation steps, the DCX-loaded LPHNp-anti-Tn antigen formulations were successfully obtained.

The attachment of the antibody to the nanoparticles was confirmed through an indirect ELISA combined with fluorescence measurements. Initially, ELISA plates were sensitized with the asialo ovine submaxillary mucin (aOSM), which contains the Tn antigen, and were incubated with the fluorescent LPHNp-Coumarin-ChiTn mAb. Subsequently, incubation with an HRP-conjugated secondary antibody was performed. Fluorescence intensity measurements confirmed the presence of the nanoparticles, while the binding of the secondary antibody demonstrated that the ChiTn mAb maintained its functionality after attaching to the nanoparticles.

### 2.5. Nanoparticle Characterization

#### 2.5.1. Particle Size and Zeta Potential

The hydrodynamic diameter and zeta potential of the samples were determined using a particle size/zeta potential analyzer (Nano-ZS, Malvern Instruments Ltd., Malvern, Worcestershire, UK). Measurements were performed in an aqueous medium at 25 °C, with a viscosity of 0.8872 cP and a refractive index of 1.33. Particle size was evaluated using dynamic light scattering (DLS) and reported as the z-average, while zeta potential was calculated from the electrophoretic mobility using the Helmholtz–Smoluchowski equation. Each sample was analyzed in triplicate, and the results are presented as mean ± standard deviation (S.D.).

#### 2.5.2. Surface Morphology

The morphology of the nanocarriers was examined using high-resolution transmission electron microscopy (HRTEM) with an HR-STEM JEOL 2100 microscope (Jeol Ltd., Tokyo, Japan). The samples were stained with a 2% (*w*/*v*) uranyl acetate solution, placed on a carbon film-covered copper grid (300-mesh), and air-dried for 24 h before scanning.

#### 2.5.3. Raman Spectroscopy 

The formation of hybrid nanoparticles was examined using confocal Raman spectroscopy, performed with a WITec Alpha 300-RA Raman-Confocal Microscope (WITec GmbH, Ulm, Germany). PLGA, DOTAP, DSPE-PEG, DSPE-PEG-Biotin, crodamol, and LPHNp were analyzed. All the samples were placed on a silicon wafer and the nanoparticle aqueous suspension was dried under a stream of nitrogen before measurement.

#### 2.5.4. Atomic Force Microscopy

LPHNps were examined through atomic force microscopy (AFM) in tapping mode using a WITec Alpha 300-RA AFM Microscope (WITec GmbH, Ulm, Germany). The AFM data were acquired by depositing a droplet of the nanoparticle suspension onto a silicon wafer substrate, and the sample was subsequently dried under a stream of nitrogen.

#### 2.5.5. Encapsulation Efficiency of DCX-Loaded Nanoparticles

The encapsulation efficiency (EE%) of DCX within LPHNp was calculated indirectly by subtracting the amount of free drug in the supernatant from the total amount of DCX in the formulation. For this process, the nanoparticles were centrifuged in Amicon tubes (10 KDa) at 4000× *g* for 15 min, and the concentration of free DCX was measured using high-performance liquid chromatography (HPLC). The HPLC system consisted of a Thermo Scientific Ultimate 3000, equipped with a UV detector set at 232 nm, and coupled with a reverse-phase Zorbax Extend 300 C18 column (4.6 mm × 150 mm i.d., 3.5 μm pore size, Agilent, Santa Clara, CA, USA). The standard calibration curves for DCX demonstrated excellent linearity (r^2^ > 0.999) across a concentration range of 0.1–1.4 μg/mL.

#### 2.5.6. In Vitro Drug Release Study

The in vitro release profile of DCX from the nanoparticles was determined using a dynamic dialysis method. In summary, 0.5 mL of the sample was placed into a dialysis bag (cutoff 2 KDa) and dialyzed against PBS (pH 7.4) and acetate buffer (pH 5), both containing 0.5% Tween-80. This setup simulated physiological conditions and intracellular release behaviors. The dialysis bag was stirred at 120 rpm, and the temperature was kept constant at 37 °C. At specified time intervals, 1 mL of dialysate was withdrawn and immediately replaced with a fresh medium to ensure sink conditions. The drug concentration was then analyzed using HPLC.

#### 2.5.7. Short-Term Stability Testing 

Stability is an essential aspect that influences the performance and efficacy of LPHNp formulations. For stability evaluation, three batches from each group (blank LPHNp, LPHNp-DCX, and LPHNp-DCX-Chi-Tn mAb) were kept at 4 °C for 27 days. DLS was employed to assess stability, with particle size measurements taken at specified time intervals.

### 2.6. In Vitro Cell Test

#### 2.6.1. Cell Uptake Studies

The A549 cells were grown on coverslips within a 24-well plate at a density of 1 × 10^5^ cells/well for 24 h. Subsequently, the medium was replaced with the treatment solution: LPHN-Coumarin and LPHN-Coumarin-ChiTn mAb. After a 30 min incubation, the cells were washed twice with PBS and fixed with 4% paraformaldehyde. The nuclei were stained with DAPI (4′,6-diamidino-2-phenylindole) at a concentration of 1 μg/mL for 1–2 min at room temperature, followed by washing. The cells were then mounted and examined using a Zeiss LSM 800 (Zeiss, Oberkochen, Germany) confocal laser scanning microscope. For flow cytometry internalization studies, the same protocol as mentioned above was followed. The A549 cells were collected, centrifuged at 300× *g* for 10 min, and re-suspended in PBS (pH 7.4). The samples were acquired using a BD Accuri C6 Flow Cytometer (BD Biosciences, San Jose, CA, USA).

#### 2.6.2. Cell Viability Assay

The cells were plated in a 96-well plate at a density of 5 × 10^3^ cells/well. Once they reached confluence, 10 µL of the treatments—DCX, LPHNp-DCX, LPHNp-DCX-Chi-Tn mAb, and blank LPHNp—were added at various concentrations (0.19, 0.39, 0.78, 1.56, 3.125, 6.25, and 12.5 µg/mL). After 72 h of incubation, 10 µL of CCK-8 solution was introduced to each well, followed by an additional 1 h incubation at 37 °C. The absorbance was then measured at 450 nm using a microplate reader. Cell viability was calculated as a percentage compared to the control (untreated) cells.

### 2.7. In Vivo Studies

All the experiments involving animals were conducted in accordance with the local regulations and were approved by the Ethics Committee on Animal Use of Institut Pasteur of Montevideo. A total of 25 female mice (Mus musculus *nude*) aged eight to ten weeks were injected subcutaneously with 1 × 10^6^ A549 tumor cells in the left flank. To monitor progression, the tumors were measured every 3 days using a caliper, and tumor volume was calculated using the formula V = (D·d^2^)/2, where D and d represent the longest and shortest tumor diameters, respectively. Once the tumors reached a volume of approximately 100 mm^3^, the mice were randomized into five treatment groups (n = 4–5 per group). The experimental groups received intraperitoneal injections of PBS as a negative control, free DCX (Taxotere), empty LPHNp–ChiTn mAb, LPHNp-DCX, and LPHNp-DCX-ChiTn mAb, each at a dose of 10 mg DCX/kg. Treatments were administered three times every four days. The endpoint of the study was set at 100 days post-tumor cell inoculation or when the tumor volume reached 1500 mm^3^, whichever occurred first. At this stage, the animals were euthanized using 100% CO_2_.

### 2.8. Statistical Analysis 

The in vitro experiments were conducted in triplicate, and the results are presented as mean ± SD. The quantitative values were analyzed using one- and two-way analysis of variance (ANOVA), followed by post hoc tests including Tukey and Bonferroni. The tumor volume analysis involved one-way ANOVA followed by Tukey’s post-test. Survival analyses were performed using the Log-rank (Mantel–Cox) test. The Prism software package (version 8.00; GraphPad Software, San Diego, CA, USA) was used to perform the statistical tests. Significant differences are denoted by asterisks (*, *p* < 0.05; **, *p* < 0.01; ***, *p* < 0.001; ****, *p* < 0.0001).

## 3. Results

### 3.1. Synthesis and Characterization of Nanoparticles

In the current study, we employed PLGA as a representative hydrophobic polymer to serve as the foundational polymeric core for the nanoparticles, enabling the efficient encapsulation of a substantial quantity of DCX. Additionally, we introduced PEG conjugated to DSPE as a dual-purpose strategy: firstly, to extend the circulation lifespan of the nanoparticles within the bloodstream, and secondly, to act as a ligand for surface functionalization of the nanoparticles. Lastly, we utilized DOTAP-Cl as a prototypical lipid compound, facilitating the formation of a lipid monolayer at the interface between the PLGA core and the PEG shell. Since the FDA has approved PLGA and DSPE-PEG for medical applications, and DOTAP is a synthetic lipid with extensive preclinical and clinical trials toxicity data [22], we expect the LPHNp obtained should be biocompatible, biodegradable, and potentially safe as a drug carrier for clinical use.

The LPHNps were prepared using a modified version of the single-step nanoprecipitation method previously reported by Bose et al. [19], Dai et al. [20], and Klippstein et al. [21]. More specifically, the organic phase containing all the constituents, including lipids, polymers, and drugs, was gradually introduced into an aqueous phase containing a hydrophilic surfactant. The mixing of the aqueous and organic phases resulted in spontaneous emulsion, and the polymer core particles formed as a consequence of PLGA precipitation. Additionally, the DOTAP and DSPE-PEG conjugates self-assembled around the PLGA core to form a lipid monolayer covered by a PEG shell.

**Figure 1:**
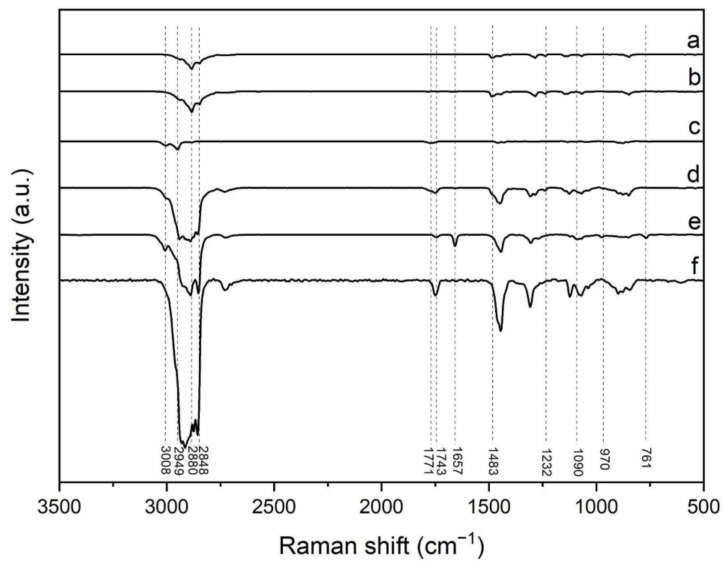
Raman spectrum of DSPE-PEG (a), DSPE-PEG-Biotin (b), PLGA (c), LPHNp (d), DOTAP-Cl (e), and crodamol (f).

To examine the intermolecular interactions inherent in the nanocomplex formation, Raman spectra were obtained from solutions comprising individual as well as combined components. In Figure 1, the Raman spectra of DSPE-PEG (a), DSPE-PEG-Biotin (b), PLGA (c), LPHNPs (d), DOTAP-Cl (e), and crodamol (f) are displayed. The most distinctive features of lipids in Raman spectroscopy are contingent upon alkyl tails. Specifically, the vibrational stretching mode of unsaturated =CH is clearly observed at 3008 cm⁻^1^, while CH_2_ Fermi resonance and symmetric stretching bands appear in the range of 2848–2880 cm⁻^1^. The C=O ester, C=C, and C–C stretching modes exhibit bands at approximately 1743 cm⁻^1^, 1657 cm⁻^1^, and 1090 cm⁻^1^, respectively. CH_2_ scissoring and twisting and =C–H in-plane deformation are situated in the range of 1232–1483 cm⁻^1^ [23,24]. In the case of DOTAP, additional bands appear at around 970, and 761 cm⁻^1^, corresponding to the trimethylammonium group [25]. In the case of PLGA, based on previous works, the peaks detected are ascribable to the vibrational modes of the lactic (LA) or/and glycolic (GA) units. There are characteristic peaks for CH_2_ (2880 cm^−1^), CH_3_ (2949 cm^−1^), and carbonyl (C=O, 1771 cm^−1^) bonds [26].

Discrepancies in the characteristic peaks previously defined for PLGA and lipids were discernible in the Raman spectra of LPHNp. These outcomes corroborated the establishment of a PLGA-lipid complex, facilitated by the hydrophobic interaction of alkyl tails and/or ester group of the hydrophobic features, as well as the ionic interactions between the carboxylic groups of PLGA and the trimethylammonium group of DOTAP.

LPHNp exhibited a monodisperse population with an average particle size of less than 250 nm and a significantly high positive surface charge (Table 1). Positively charged nanoparticles have demonstrated greater cellular internalization efficiency compared to neutral or negatively charged nanoparticles [27,28]. However, they face significant stability challenges in biological fluids due to their interactions with negatively charged biomolecules, which can lead to aggregation, protein corona formation, and rapid clearance. In our study, the incorporation of PEGylated lipids onto the surface of LPHNp addresses these challenges by providing a steric barrier that reduces unspecific interactions with plasma proteins and cells, as supported by the literature [29,30,31]. The positive charge of these nanoparticles arises from the cationic lipid monolayer coating the hydrophobic polymeric core composed of PLGA and crodamol oil. Surface charge plays a pivotal role in determining the stability of colloidal nanoparticle systems, with repulsive forces between similarly charged nanoparticles enhancing stability.

DCX, a highly water-insoluble compound, exhibits a strong affinity for the hydrophobic core of these LPHNp. This feature enables a remarkable encapsulation efficiency (EE) of 99.92% for DCX, resulting in a final loading of 4 mg DCX/mL, which is much higher than the encapsulation efficiency reported by other groups for PLGA nanoparticles. For instance, Gupta et al. [32] developed DCX-loaded PEGylated PLGA nanoparticles loaded with an average size of 150 nm and an EE of 70%. Meanwhile, Zhang et al. [33] prepared LPHNp using PLGA and lecithin, also loaded with DCX, but achieved an EE of less than 60%. Both studies utilized a total DCX concentration significantly lower than that used in our system. Achieving high encapsulation efficiency is critical in drug delivery systems, as it reduces the amount of nanoparticles needed to deliver specific doses of active agents. This strategy not only minimizes potential nanoparticle-related side effects but also enhances therapeutic efficacy and lowers overall production costs.

To produce LPHNp functionalized with Chi-Tn mAb, our focus was on the non-covalent interaction inherent in avidin–biotin binding. In this process, LPHNps, which present a biotin motif on their surface, were initially incubated with tetrameric avidin, followed by subsequent incubation with biotinylated Chi-Tn mAb. This strategy, with documented advantages in prior studies [34,35], provides precise control over the placement and density of the ligand on the nanoparticle surface, ensuring efficient ligand–receptor interactions. As expected, the encapsulation of DCX and the functionalization with the Chi-Tn antibody had little effect on the size and charge of the blank LPHNp.

**Figure 2:**
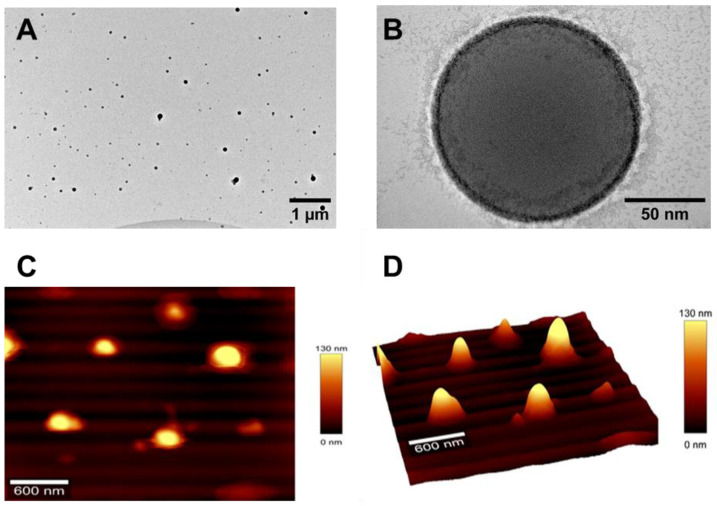
Morphological analysis of LPHNp-DCX-Chi-Tn using HR-TEM (**A**,**B**) and AFM (**C**,**D**).

The morphology of the nanosystems was examined using high-resolution transmission electron microscopy before staining with 2% uranyl acetate solution, which stains lipid structures to enhance their electron density. The TEM images of LPHNp-DCX-Chi-Tn mAb are shown in Figure 2. The lipid/polymer nanocarriers exhibit a spherical shape and a size close to 200 nm (Figure 2), a result that corresponds to that observed by DLS. Notably, this size falls within the optimal range of 70 to 300 nm, as reported in the literature, which is considered ideal for enhancing biological performance [36,37]. The thickness of the lipidic ring surrounding the PLGA core is less than 5 nm, which could correspond to a lipid monolayer plus PEG shell.

LPHNps were further examined by AFM to provide more information about surface morphology and particle size. In Figure 2, both the 2D and 3D AFM images illustrate alterations in surface topography, indicating the presence of nanoparticles with a spherical shape. A preliminary assessment of the peaks from the AFM topography analysis revealed that the nanoparticles exhibited a particle size of 250 nm, aligning with the data obtained from the TEM and DLS.

### 3.2. In Vitro Drug Release and Storage Stability Test

**Figure 3:**
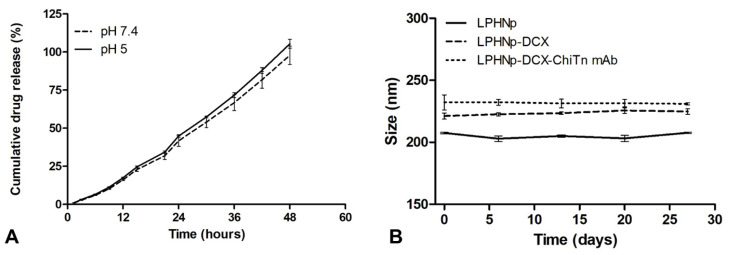
In vitro drug release of DCX from LPHNp-DCX-Chi-Tn mAb in buffer at pH 7.4 and 5 at 37 °C (**A**). Size distribution of LPHNp during 27 days of storage at 4 °C (**B**).

The in vitro release profile of DCX from the hybrid nanoparticles under different conditions, pH 5 (which mimics the acidic environment found in the endosomes or tumor microenvironment) and pH 7.4 (representing the physiological conditions in the blood), is illustrated in Figure 3A. The presence of Tween-80 in the buffer solution can facilitate the release of DCX from the matrix polymer, as previously reported [38,39,40]. The nanoparticles exhibited sustained drug release behavior for up to 48 h, with a nearly identical pattern in both pH media but higher at pH 5 compared to pH 7.4. The release kinetics followed a zero-order model with a correlation coefficient (r^2^) of 0.9915 for pH 7.4 and 0.9877 for pH 5.

The stability of nanocarriers is a crucial factor for their storage and functionality in long-term biomedical applications. To assess the shelf life of the formulations, three batches of each group—LPHNp, LPHNp-DCX, and LPHNp-DCX-Chi-Tn mAb—were stored at 4 °C. The z-average, representing the average intensity-weighted diameters, was employed to investigate the stability of LPHNps through dynamic light scattering (DLS) measurements at various time intervals. As depicted in Figure 3B, all the samples stored at 4 °C remained stable for 27 days, exhibiting a uniform appearance without any visible signs of instability, such as sedimentation or aggregation.

### 3.3. Cell Uptake Study

**Figure 4:**
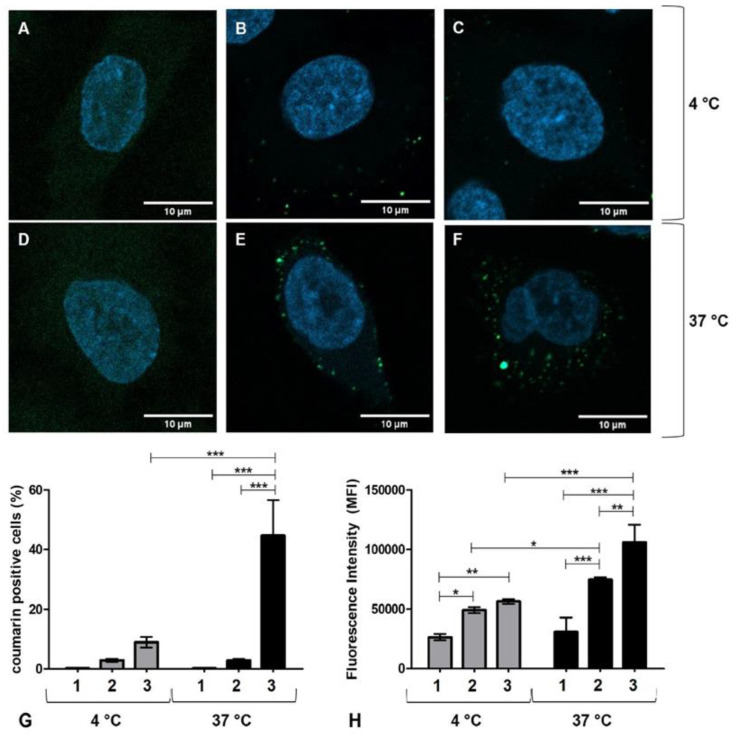
Uptake study of fluorescent LPHNp in A549 cancer cells. (**A**–**F**) Images obtained by CLSM of untreated cells (**A**,**D**), LPHN-coumarin (**B**,**E**), and LPHN-coumarin-ChiTn mAb (**C**,**F**) incubated at 4 °C (**A**–**C**) or 37 °C (**D**–**F**). (**G**) Percentage of 6-coumarin positive cells evaluated by flow cytometry. Numbers 1, 2, and 3 represent untreated cells, LPHN-coumarin-treated cells, and LPHN-coumarin-ChiTn mAb-treated cells, respectively. (**H**) Mean fluorescence intensity (MFI) of untreated cells (1), LPHN-coumarin-treated cells (2), and LPHN-coumarin-ChiTn mAb-treated cells determined by flow cytometry. Significant differences between groups are indicated by asterisks: * represents *p* < 0.05, ** represents *p* < 0.01, and *** represents *p* < 0.001.

In order to study the uptake of LPHNp by the A549 cancer cell line, a fluorescent dye was encapsulated, and its uptake was observed using confocal microscopy. For this purpose, 6-coumarin was loaded into LPHNp, with or without the Chi-Tn mAb, and then incubated at 37 °C or 4 °C as a negative control for endocytosis. As shown in Figure 4, the confocal microscopy images revealed that at 4 °C, the nanoparticles were primarily located outside the cell or associated with the cell surface (Figure 4B,C). In contrast, at 37 °C, the LPHNps were fully internalized by A549, appearing to be within vesicles (Figure 4E,F). This indicates that the encapsulated dye was likely internalized by the cell through an endocytic mechanism. Additionally, the images showed that the association of 6-coumarin LPHNps with A549 cells seems to be enhanced by the presence of Chi-Tn mAb.

Flow cytometry supported the qualitative findings of the CLSM studies. The results demonstrated that the A549 cell line efficiently internalized LPHNps, leading to an increase in both the percentage of 6-coumarin-LPHNp+ cells (Figure 4G) and 6-coumarin-LPHNp mean fluorescence intensity (MFI) (Figure 4H) compared to the untreated cells and A549 cells incubated at 4 °C, serving as a control to minimize active internalization. This finding confirms that the Chi-Tn antibody enhances cellular internalization, these findings align with the prior results from our group, where Chi-Tn mAb demonstrated rapid internalization across multiple cancer cell lines and was observed within early and recycling endosomes [17].

### 3.4. Cell Viability Assay

**Figure 5:**
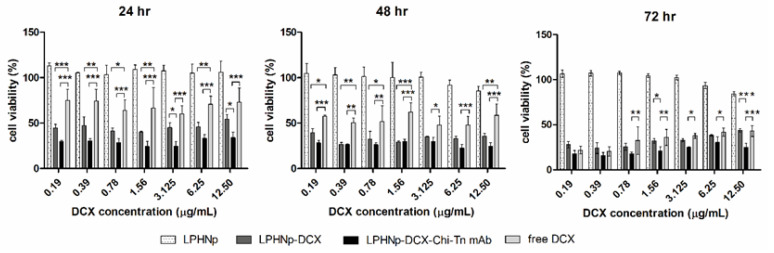
Cell viability was assessed in the A549 cell line using the CCK-8 assay for free DCX and DCX-loaded LPHNp formulations at varying concentrations over 24, 48, and 72 h. Significant differences are denoted by asterisks (*, *p* < 0.05; **, *p* < 0.01; ***, *p* < 0.001).

To evaluate the cytotoxicity of the obtained LPHNps and free DCX, a CCK-8 colorimetric cell viability assay was performed on the A549 human lung cancer cell line. The cells were incubated with LPHNp-DCX, LPHNp-DCX-Chi-Tn, free DCX, and blank LPHNp for 24, 48, and 72 h at concentrations ranging from 0.19 to 12.5 µg/mL. The results, depicted in Figure 5, demonstrate that LPHNps loaded with DCX significantly reduce the cell viability of the A549 cells compared to free DCX at 24 and 48 h of incubation. However, this difference becomes less pronounced at 72 h. This may be due to the accelerated uptake of the encapsulated DCX in the nanosystems and the consequent release of the drug into the cytosolic space. Moreover, the Chi-Tn antibody-functionalized LPHNps further enhances this antiproliferative response in the A549 cells, showing a significant difference compared to both LPHNp-DCX and free DCX. This suggests that the antibody enhances the internalization of the nanosystems, an observation supported by cellular uptake studies. Conversely, blank LPHNp carriers showed no impact on cell growth.

### 3.5. In Vivo Anti-Tumor Activity

**Figure 6:**
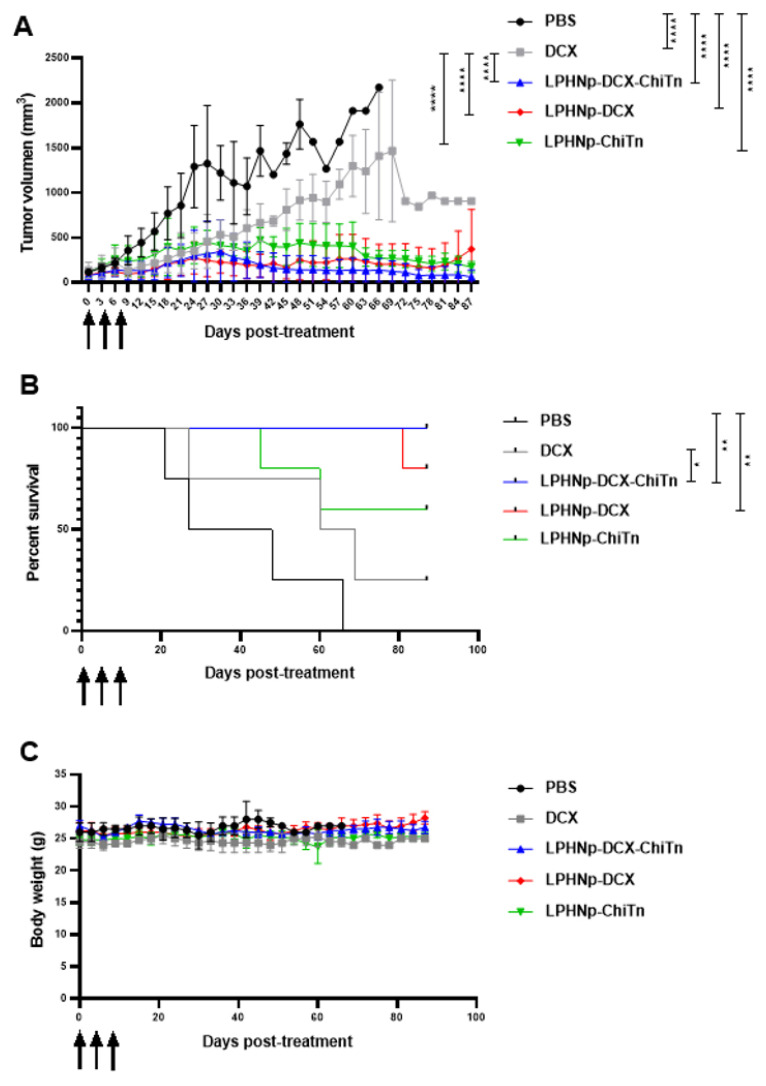
Anti-tumor effect in vivo. *Nude* mice were inoculated with the A549 lung tumor cell line. When the tumors reached approximately 100 mm^3^, the animals were treated intraperitoneally with different treatments: PBS, free DCX (Taxotere), LPHNp-DCX-Chi-Tn, LPHNp-DCX, and 6, LPHNp-Chi-Tn, each formulation at 10 mg DCX/kg. The treatment was administered three times every four days. The black arrows indicate the days on which treatment was given. (**A**) Inhibition of lung tumor xenograft growth in the *nude* mice by the nanoparticle formulations. Data are presented as mean ± SD. (**B**) Kaplan–Meier survival curve of the treated mice. (**C**) Body weight was measured at the indicated times. Data are presented as mean ± SD. Significant differences are denoted by asterisks (*, *p* < 0.05; **, *p* < 0.01; ****, *p* < 0.0001).

In order to evaluate the anti-tumor effects of DCX loaded in Chi-Tn mAb-functionalized LPHNp in an in vivo model, A549 human lung cancer cells were subcutaneously injected into the flank of *nude* mice to establish xenotransplants. The animals were then subjected to various treatments: PBS (control group), free DCX, empty LPHNp–Chi-Tn mAb, LPHNp-DCX, and LPHNp-DCX-Chi-Tn mAb, administered in three doses of 10 mg DCX/kg each, every four days. The tumor volume and survival rate were measured to evaluate the impact of the different treatments. As expected, the animals treated with PBS exhibited the most significant tumor growth and the lowest survival rate (Figure 6A,B). Conversely, the animals treated with LPHNp-DCX-Chi-Tn displayed the least tumor growth and the highest survival rate. In addition, both LPHNp-DCX and LPHNp-Chi-Tn also decreased tumor growth similarly to LPHNp-DCX-Chi-Tn; indeed, no significant differences were observed among these treatment groups. Furthermore, all the nanoparticle formulations demonstrated a significant decrease in tumor growth compared to the commercial Taxotere formulation (free DCX), resulting also in a higher survival rate. However, the unique treatment that significantly enhanced the survival rate compared to DCX was the LPHNp-DCX-Chi-Tn preparation. None of the formulated preparations had any observed adverse effects on the body weight of the mice, indicating that the dose of DTX or the formulated preparations administered were well tolerated (Figure 6C). The improved efficacy of the encapsulated DCX could be attributed to the rapid internalization of the nanoparticles, facilitating intracellular drug delivery, which is in agreement with the in vitro cytotoxicity studies. However, anti-tumor activity is also observed in LPHNp-Chi-Tn, an effect that is logically attributed to the Chi-Tn antibody. Previous research has demonstrated that this antibody triggers antibody-dependent cellular cytotoxicity (ADCC) [16]. This implies that the Chi-Tn antibody does not directly exert cytotoxic effects on tumor cells in vitro or in vivo; instead, its action is based on activating the immune system to attack tumor cells. By recognizing the Tn antigen present in tumor cells, this antibody flags them for destruction by effector immune cells possessing Fc receptors (FcγR), such as macrophages, neutrophils, and NK cells. It is worth noting that the mice used as animal models in this study are immunocompromised due to genetic mutations affecting thymic functionality, resulting in a lack of mature T cells. However, they retain other components of the immune system, primarily macrophages and NK cells, which may contribute to the observed anti-tumor response [41]. These findings suggest that LPHNp could serve as excellent vehicles for drug delivery and release at the target site, either through passive targeting mechanisms exploiting the enhanced permeability and retention effect (EPR) or through active targeting via conjugation with the Chi-Tn mAb, which can also enhance the anti-tumor response via the ADCC mechanism.

## 4. Conclusions

In this study, we developed DCX-loaded LPHNp functionalized with the Chi-Tn antibody, proposing it as an innovative active targeting drug delivery system for lung cancer therapy. The implementation of LPHNp achieved nanocarriers with a high DCX content, exhibiting sustained drug release kinetics. Moreover, DCX-loaded LPHNps-Chi-Tn mAb can internalize into the A549 cell cytoplasm and decrease cell viability more efficiently than non-functionalized nanoparticles or the free drug. Furthermore, the nanoparticle formulations demonstrated more effective anti-tumor activity in vivo compared to the commercial Taxotere formulation (free DCX), resulting in a higher survival rate. These results suggest that LPHNp-Chi-Tn mAb could be utilized as active targeting nanocarriers with controlled drug release to enhance oncological treatments and minimize secondary effects.

## Figures and Tables

**Table 1:** Physicochemical properties of the nanosystems obtained (mean ± S.D.; n = 3).

Formulation	Size ± SD (nm)	IP ± SD	Z Potential ± SD (mV)	EE ± SD (%)
LPHNp	201.9 ± 2.3	0.1 ± 0.01	32.1 ± 1.2	-
LPHNp-Chi-Tn mAb	205.5 ± 1.5	0.2 ± 0.01	36.6 ± 1.6	-
LPHNp-DCX	222.5 ± 1.3	0.1 ± 0.02	36.0 ± 0.9	99.92 ± 0.01
LPHNp-DCX-Chi-Tn mAb	232.3 ± 2.4	0.2 ± 0.02	37.6 ± 0.8	99.92 ± 0.01

## Data Availability

The characterization data and experimental protocols for this work are available within this manuscript, or from the corresponding author upon request.
